# The nematicidal potential of novel fungus, *Trichoderma asperellum* FbMi6 against *Meloidogyne incognita*

**DOI:** 10.1038/s41598-023-33669-z

**Published:** 2023-04-23

**Authors:** Ritul Saharan, J. A. Patil, Saroj Yadav, Anil Kumar, Vinod Goyal

**Affiliations:** 1 Department of Nematology, CCS Haryana Agricultural University, Hisar, Haryana India; 2 Department of Botany and Plant Physiology, CCS Haryana Agricultural University, Hisar, Haryana India

**Keywords:** Microbiology, Plant sciences

## Abstract

One of the most damaging pests in vegetable crops is the root-knot nematode (*Meloidogyne incognita*) worldwide. The continuous use of nematicide is costly and has unintended consequences for human and environmental health. To minimize nematicides, eco-friendly integrated nematode management is required. *Trichoderma,* an antagonistic fungus has been explored to control root-knot nematode. The fungal bio-control strain FbMi6 was identified as *Trichoderma asperellum* (accession no. MT529846.1). *T. asperellum* FbMi6 showed substantial nematicidal activity in the laboratory, with egg hatch suppression (96.6%) and juvenile mortality (90.3%) of *M. incognita*. *T. asperellum* FbMi6 was examined under pot and field  conditions (after neem cake enrichment), both alone and in combination, and compared with controls. Application of *T. asperellum* FbMi6 enriched neem cake (1-ton ha^-1^) increased (28.3%) the okra yield and decreased (57.1%) nematode population as compared with control. *T. asperellum* FbMi6 enriched neem cake had higher polyphenol content (resistance enhancer) in okra compared with inoculated check.

## Introduction

Okra (*Abelmoschus esculentus* L.) is a popular vegetable crop produced for its health benefits in tropical and subtropical regions around the world. Southern root-knot nematode (*Meloidogyne incognita*), a phytonematode, is critical biotic stress on okra, producing substantial root damage and affecting the production value and quantity^1,2^. Okra production in India is being endangered by the spread of *Meloidogyne* spp. in all growing areas^3^. Root-knot nematode caused 19.6% yield losses in all vegetable crops in India^3^. In India, no nematicides for okra are currently suggested^4^. Although, efforts to develop resistant cultivars are being made, none are currently available^5^. As a result, biological control is suggested as a safer alternative for living biota and its environment^6^ to control the root-knot nematode in vegetables^7^. *Trichoderma*, a potential biocontrol agent, has successfully been utilized in vegetable crops to control root-knot nematodes^8–11^. *Trichoderma* spp. provides different plant health benefits, like promotion in plant growth, disease control, stress tolerance and development of resistance in plants. *Trichoderma asperellum* is an emerging and effective biocontrol agent, for its endless effectiveness in managing plant parasitic nematodes and disease complexes with other secondary pathogens^12–15^. Plant parasitic nematodes are inhibited by various *Trichoderma* species used as biocontrol agents^8,16–18^. Now, many actions of *Trichoderma* are documented as BCA: including competition, antibiosis, resistance, and plant tolerance against biotic and abiotic stresses and stimulation of its defenses against pathogens^19^. *Trichoderma* spp. has faster reproduction than other microorganisms/bioagents and has a strong acclimating capacity in a diversified environment^20,21^. *Trichoderma* spp. emit elicitors when they interact with plants, which promotes systemic acquired resistance and immunity against plant diseases and pests. Plant pathogens are inhibited by primary and secondary metabolites generated by *Trichoderma* spp. Many secondary metabolites generated by *Trichoderma* spp., such as flavonoids and phenols offer resistance to biotic stress^22,23^. Keeping in view, the present study was conducted on indigenous *T. asperellum* FbMi6 strain performed against *M. incognita* under in vitro and in vivo conditions in okra.

## Results

### Morphological and molecular identification of isolate FbMi6

**Figure 1:**
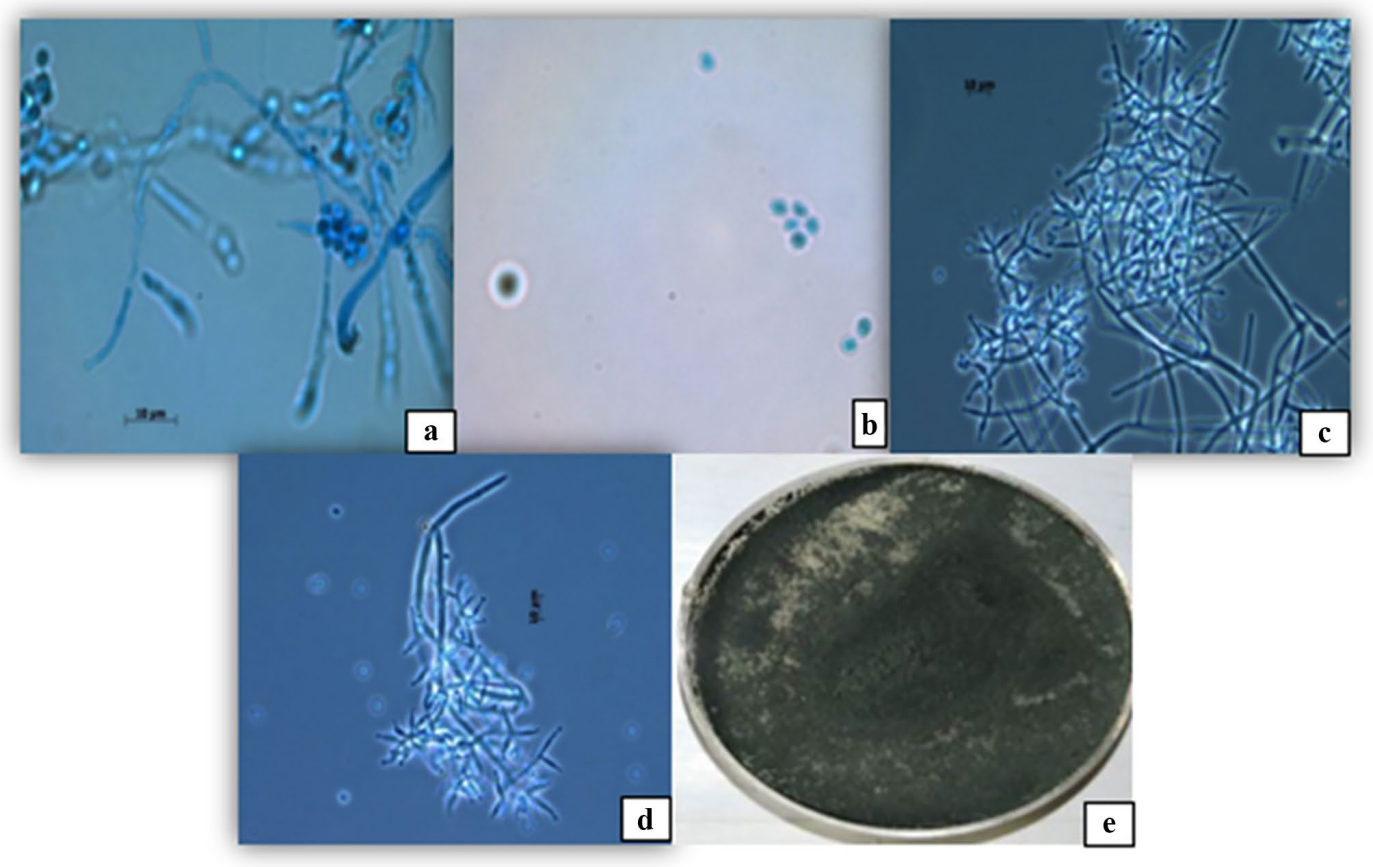
Microscopic characters of *Trichoderma asperellum* (**a**) conidiophore, (**b**) conidia (**c**), hyphae, (**d**) fialida, (**e**) colonial morphology of FbMi-6 on PDA medium.

**Figure 2:**
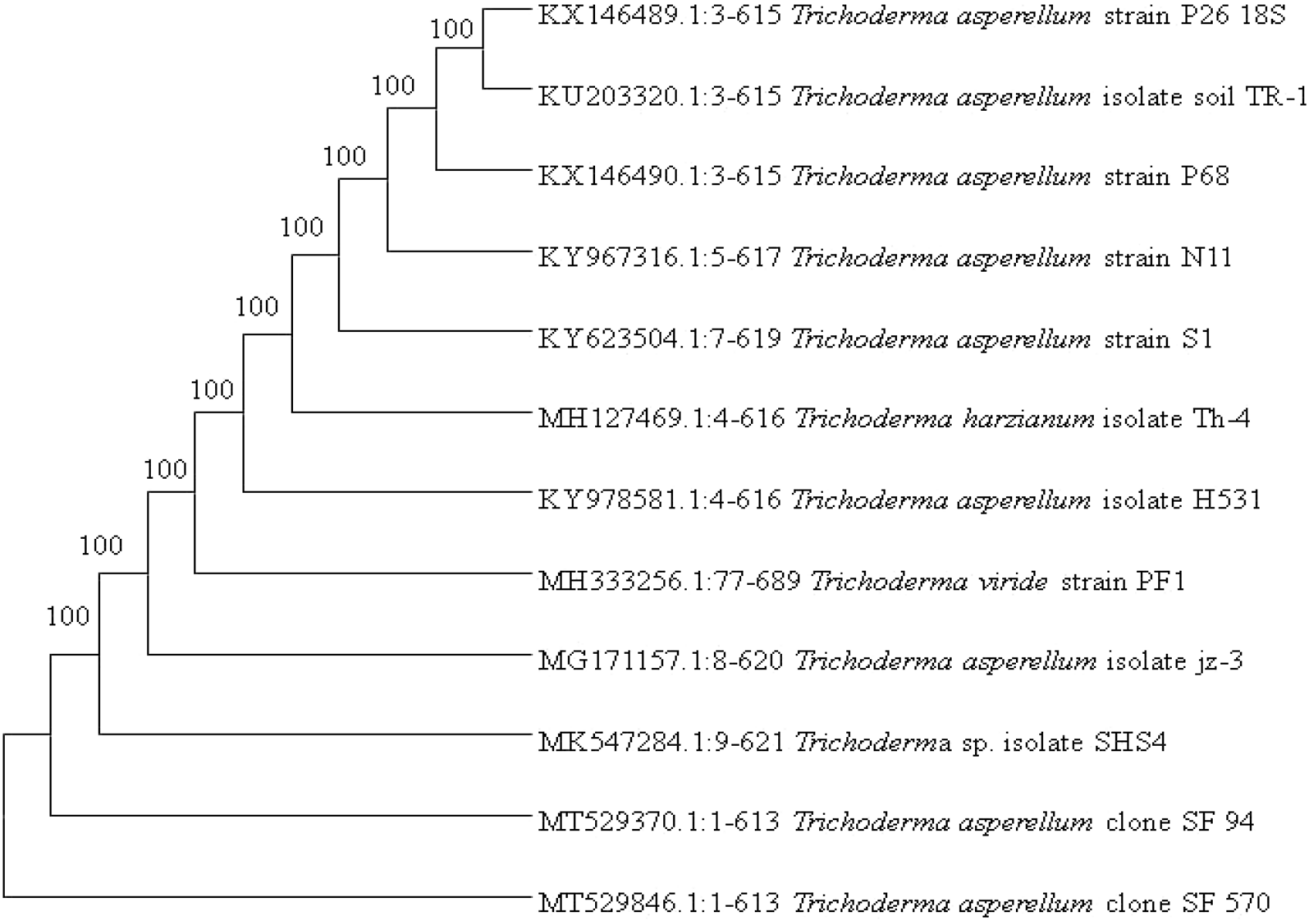
Phylogenetic tree of *T. asperellum* FbMi6 based on ITS-rDNA gene sequences. In-vitro efficacy of bio-agent.

At 7 days, morphological observations of FbMi6 revealed that colonies with fluffy mycelial development and a large conidial zone at the colony's periphery, following sporulation, whitish or yellowish growth turned greenish or pale greenish features were identified as *Trichoderma* (Fig. 1). Morphological features of isolate FbMi-6, such as hyphae up to 7.45 µm broad, conidiophore with a maximum length of 3.2 × 197.3 µm, chlamydospores with a diameter of up to 9 × 10.4 µm, phialides range in size from 1.7–2.8 × 9.0–16.5 µm and phialospores with a diameter of 2.7–3.2 × 3.4–4.4 µm were measured (Fig. 1). Based on morphological and molecular characteristics, isolate FbMi6 was identified as *T. asperellum*. The sequence was submitted to GenBank (NCBI), with accession number MT529846.1. It showed 100% similarity to *T. asperellum* as per GenBank data. Also, according to phylogenetic analysis, FbMi6 was found most closely related to *T. asperellum* (Fig. 2).

**Figure 3:**
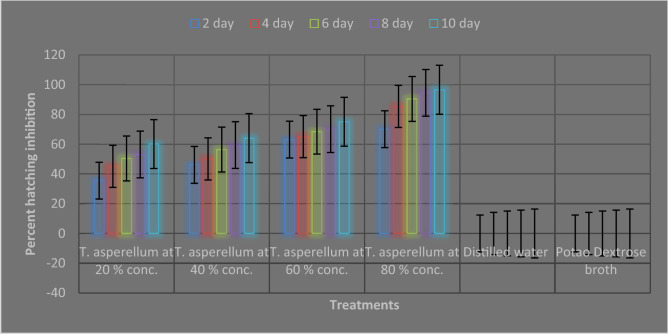
Effect of *T. asperellum* FbMi6 on egg hatching inhibition of *M. incognita* under in-vitro conditions.

In comparison with the control, *T. asperellum* FbMi6 significantly reduced hatchability and having maximal juvenile mortality. *T. asperellum* FbMi6 inhibited egg hatching in all concentrations as compared to control. At 120 h, maximum egg hatching inhibition (96.6%) was observed at 80% concentration of *T. asperellum* FbMi6 as compared with control. With increasing exposure period, egg hatching inhibition gradually increased. *T. asperellum* FbMi6 culture filtrates cause egg deformation and hatching suppression starting at 24 h, in all the concentrations (Fig. 3).

**Figure 4:**
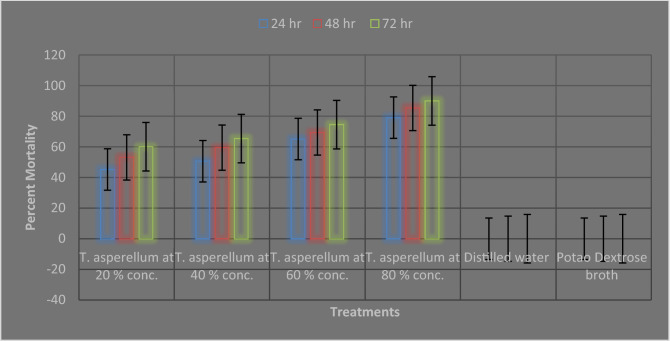
Effect of *T. asperellum* FbMi6 on juveniles (J_2_) mortality of *M. incognita* under in-vitro conditions.

Similarly, *T. asperellum* FbMi6 culture filtrate showed antagonistic or nematocidal activity against *M. incognita* juveniles in all the concentrations (Fig. 4). *T. asperellum* FbMi6 showed maximum root-knot nematode juveniles’ mortality in all concentrations at 72 h exposure as compared to control. *T. asperellum* FbMi6 at 80% concentration had the highest (90.3%) juvenile mortality compared to control. The mortality of juveniles enhanced as exposure time increased (Fig. 4).

### Effects of *T. asperellum* FbMi6 enriched neem cake on *M. incognita* and vegetative growth of okra

The application of *T. asperellum* FbMi6 enriched neem cake had substantial (*P* ≤ 0.05) suppressive effects on *M. incognita* population in terms of J_2_s 200^–1^ cc soil, galls and egg masses plant^−1^, and eggs per egg mass, as well as promote the vegetative growth of okra. The gall index and egg masses were significantly reduced with *T. asperellum* FbMi6 enriched neem cake (Table 1). Fewer galls on okra roots were observed with *T. asperellum* FbMi6 enriched neem cake at 20 g kg^−1^ soil. Egg masses and eggs per egg mass were also lower in all *T. asperellum* FbMi6 enriched neem cake treatments compared with control. In comparison to the untreated inoculated check, soil nematode population was considerably reduced in all the treatments of *T. asperellum* FbMi6 enriched neem cake. In contrast to the untreated control, *T. asperellum* FbMi6 enriched neem cake had the lowest nematode population at 20 g kg^−1^ soil application.

**Table 1:** Efficacy of *T. asperellum* FbMi6 enriched neem cake on nematode reproduction parameters of *M. incognita* in okra.

Treatment	Galls plant^−1^	Egg masses plant^−1^	Eggs egg mass^−1^	J_2_ 200^–1^ cc soil
TNC at 5 g kg^−1^ soil	86 $\pm$ 1.0^f^	67 $\pm$ 0.8^f^	291 $\pm$ 1.1^f^	530 $\pm$ 4.3^f^
TNC at 10 g kg^−1^ soil	79 $\pm$ 1.0^e^	59 $\pm$ 1.3^e^	248 $\pm$ 1.3^e^	379 $\pm$ 15.2^e^
TNC at 15 g kg^−1^ soil	63 $\pm$ 1.6^d^	47 $\pm$ 1.5^d^	224 $\pm$ 2.4^d^	341 $\pm$ 3.9^d^
TNC at 20 g kg^−1^ soil	54 $\pm$ 1.1^c^	39 $\pm$ 1.3^b^	189 $\pm$ 0.8^c^	199 $\pm$ 6.0^c^
Carbofuran 3G at 16 mg kg^−1^ soil	40 $\pm$ 0.8^b^	44 $\pm$ 0.8^c^	184 $\pm$ 1.1^b^	162 $\pm$ 1.1^b^
Untreated inoculated check	163 $\pm$ 1.3^g^	96 $\pm$ 0.8^g^	432 $\pm$ 1.3^g^	982 $\pm$ 7.7^g^

Data are mean of five replicates. INP- 2000 J_2_/kg. Values with the different letter are significant according to DMRT (p < 0.05). Data are presented as means $\pm$ SD.

*TNC* *T. asperellum* FbMi6 enriched neem cake.

Plant biomass (shoot and root weight) differed significantly between *T. asperellum* FbMi6 enriched neem cake and control (untreated). When compared to the untreated inoculated control, all treatments with *T. asperellum* FbMi6 enriched neem cake significantly enhanced plant height (*P* ≤ 0.05). The results (Table 2) showed that *T. asperellum* FbMi6 enriched neem cake at 20 g kg^−1^ soil had considerably higher plant height than *T. asperellum* FbMi6 enriched neem cake at 15 g kg^−1^ soil. At increasing doses, *T. asperellum* FbMi6 enriched neem cake generated the maximum dry root and shoot weight.

**Table 2:** Efficacy of *T. asperellum* FbMi6 enriched neem cake on plant growth parameters of okra infected with *M. incognita*.

Treatment	Plant height (cm)	Fresh root wt. (g)	Fresh shoot wt. (g)	Dry shoot wt. (g)
TNC at 5 g kg^−1^ soil	22.80 $\pm$ 0.6^b^	0.55 $\pm$ 0.1^a^	4.86 $\pm$ 0.1^b^	0.90 $\pm$ 0.0^bc^
TNC at 10 g kg^−1^ soil	24.46 $\pm$ 0.6^bc^	1.88 $\pm$ 0.0^d^	5.32 $\pm$ 0.1^c^	1.04 $\pm$ 0.0^bd^
TNC at 15 g kg^−1^ soil	27.84 $\pm$ 0.6^d^	1.23 $\pm$ 0.1^c^	8.50 $\pm$ 0.3^e^	1.70 $\pm$ 0.0^be^
TNC at 20 g kg^−1^ soil	35.30 $\pm$ 3.8^e^	2.57 $\pm$ 0.1^e^	11.86 $\pm$ 0.3^f^	3.48 $\pm$ 0.0^bg^
Carbofuran 3G at 16 mg kg^−1^ soil	27.60 $\pm$ 0.8^d^	0.64 $\pm$ 0.1^a^	6.92 $\pm$ 0.3^d^	0.80 $\pm$ 0.0^bb^
Untreated uninoculated check	26.36 $\pm$ 0.4^cd^	1.22 $\pm$ 0.1^c^	7.14 $\pm$ 0.0^d^	1.86 $\pm$ 0.0^f^
Untreated inoculated check	19.60 $\pm$ 0.7^a^	0.85 $\pm$ 0.0^b^	3.34 $\pm$ 0.1^a^	0.52 $\pm$ 0.1^a^

Data are mean of five replicates. INP- 2000J_2_/kg. Values with the different letter are significant according to DMRT (p < 0.05). Data are presented as means $\pm$ SD.

*TNC* *T. asperellum* FbMi6 enriched neem cake.

### Effect of *T. asperellum* FbMi6 enriched neem cake on biochemical and physiological parameters of okra infested with *M. incognita*

According to the results (Table 3), *T. asperellum* FbMi6 enriched neem cake at 20 g kg^−1^ soil had a considerably higher nitrogen balance index than *T. asperellum* FbMi6 enriched neem cake at 15 g kg^−1^ soil, while untreated inoculated check had the lowest nitrogen balance index. In comparison to the untreated inoculated control, all treatments of *T. asperellum* FbMi6 enriched neem cake had significantly higher total chlorophyll content. *T. asperellum* FbMi6 enriched neem cake at 20 g kg^−1^ soil had considerably higher anthocyanin content than the untreated inoculated control and rest of the treatments were non-significant. *T. asperellum* FbMi6 enriched neem cake at 20 g kg^−1^ soil had the highest polyphenol content, followed by *T. asperellum* FbMi6 enriched neem cake at 15 g kg^−1^ soil. When compared to the untreated inoculated control, all treatments exhibited considerably higher flavonoid content. The highest flavonoid concentration was recovered in *T. asperellum* FbMi6 enriched neem cake at 20 g kg^−1^ soil, while the lowest was recorded in the untreated inoculated control.

**Table 3:** Efficacy of *T. asperellum* FbMi6 enriched neem cake on biochemical and physiological parameter of okra infected with *M. incognita*.

Treatment	NBI	Total chlorophyll	Anthocyanin	Polyphenol	Flavonoids
TNC at 5 g kg^−1^ soil	35.72 $\pm$ 0.7^b^	23.98 $\pm$ 0.7^a^	0.02 $\pm$ 0.0^a^	0.26 $\pm$ 0.0^d^	0.52 $\pm$ 0.0^b^
TNC at 10 g kg^−1^ soil	37.44 $\pm$ 0.5^c^	32.92 $\pm$ 0.6^c^	0.06 $\pm$ 0.0^a^	0. 25 $\pm$ 0.0^e^	0.75 $\pm$ 0.0^c^
TNC at 15 g kg^−1^ soil	40.48 $\pm$ 0.5^d^	41.62 $\pm$ 0.9^d^	0.10 $\pm$ 0.0^ab^	0.28 $\pm$ 0.0^f^	0.95 $\pm$ 0.0^d^
TNC at 20 g kg^−1^ soil	42.06 $\pm$ 0.6^e^	50.60 $\pm$ 0.8^e^	0.25 $\pm$ 0.0^a^	0.50 $\pm$ 0.0^g^	1.04 $\pm$ 0.0^f^
Carbofuran 3G at 16 mg kg^−1^ soil	30.92 $\pm$ 0.5^a^	30.10 $\pm$ 0.7^b^	0.02 $\pm$ 0.3^a^	0.17 $\pm$ 0.0^a^	1.02 $\pm$ 0.0^f^
Untreated uninoculated check	41.88 $\pm$ 0.9^e^	40.94 $\pm$ 0.4^d^	0.13 $\pm$ 0.0^ab^	0.24 $\pm$ 0.0^c^	0.98 $\pm$ 0.0^e^
Untreated inoculated check	30.60 $\pm$ 0.6^a^	23.76 $\pm$ 1.0^a^	0.02 $\pm$ 0.0^a^	0.19 $\pm$ 0.0^b^	0.34 $\pm$ 0.0^a^

Data are mean of five replicates. Values with the different letter are significant according to DMRT (p < 0.05). Data are presented as means $\pm$ SD.

*NBI* nitrogen balance index, *TNC* *T. asperellum* FbMi6 enriched neem cake.

### Effect of *T. asperellum* FbMi6 enriched neem cake on growth and yield of okra infecting root-knot nematode under field conditions

Colonies of *T. asperellum* FbMi6 culture was found to be 8 × 10^8^ CFU g^−1^ after enrichment in neem cake. In a field experiment, soil application of *T. asperellum* FbMi6 enriched neem cake resulted in significantly greater yield and lower nematode population. The carbofuran-treated plots had less nematode reproduction than those with *T. asperellum* FbMi6 enriched neem cake. *T.asperellum* FbMi6 enriched neem cake and carbofuran inhibited root galls as comparison to control. The application of *T. asperellum* FbMi6 enriched neem cake and carbofuran considerably reduced the soil nematodes population (second stage juveniles). Nonetheless, results (Table 4) demonstrated that *T. asperellum* FbMi6 enriched neem cake at 1-ton ha^−1^ boosted okra output/yield substantially as compared with control. Okra yield in *T. asperellum* FbMi6 enriched neem cake was higher than the chemical control (carbofuran @ 1.0 kg a.i. ha^−1^). *T. asperellum* FbMi6 enriched neem cake at 1-ton ha^−1^ increased 28.3% okra yield above the control.

**Table 4:** Efficacy of *T. asperellum* FbMi6 enriched neem cake in okra against *M. incognita*.

Treatments	Root gall index	Soil population (200^–1^ cc soil)	Yield (q/ha)
NC 1-ton ha^−1^	3.2 $\pm$ 0.5^c^	$215\pm$ 5.8^c^	$54.4\pm$ 5.9^a^
TNC 1-ton ha^−1^	2.6 $\pm$ 0.9^d^	184 $\pm$ 6.9^d^	60 $\pm$ 4.5^d^
Carbofuran 1 kg a.i. ha^−1^	3.8 $\pm$ 0.8^b^	233 $\pm$ 7.5^b^	50 $\pm$ 6.0^b^
Untreated control	4.8 $\pm$ 0.4^a^	429 $\pm$ 18.6^a^	43 $\pm$ 3.8^c^

Data are mean of five replicates. INP- 235 J_2_/200 cc soil. Values with the different letter are significant according to DMRT (p < 0.05). Data are presented as means $\pm$ SD.

*NC* neem cake; *TNC* *T. asperellum* FbMi6 enriched neem cake.

## Discussion

Many countries, including the United States, Australia, India, and China, have recently added to the collection of *Trichoderma* sources. *Trichoderma* research first concentrated on soil flora and fauna, soil remediation, soil biology, pollution, and general mycology. As our understanding of *Trichoderma* grew, researchers concentrated on biocontrol and its applications in plant nematology and mycology. Isolate FbMi6 was identified as *T. asperellum* based on cultural, morphological, and molecular findings in this study. *Trichoderma* species has also been found in diversified environment and isolated from the upper atmosphere^24^, wetlands^25^, and rhizosphere soil^26^. There have been 141 or more *Trichoderma* species recorded worldwide^27–30^. *T. asperellum* FbMi6 showed substantial antagonistic activity on egg hatching and juvenile mortality of *M. incognita* in present investigation, suggesting a possible nematoxic compounds in it. Many scientists have already been conducted research on *Trichoderma* isolates for nematode inhibition in vitro^31–34^. Deformation of eggs and juveniles confirmed the presence of nematicidal compounds in culture filtrates of *Trichoderma*. *T. asperellum* FbMi6 generated nematicidal chemicals that appeared to play a key role in nematode death. Plant parasitic nematode infestations have been reduced using *Trichoderma* species as biocontrol agents^35,36^. Biocontrol effectiveness of these microbes was demonstrated by the antagonism of *Trichoderma* culture filtrates against *M. incognita*. Meyer^37^observed that 253 fungal isolates (*Acremonium* sp., *Aspergillus* sp., *Fusarium* sp., *Paecilomyces* sp.) had a nematicidal influence on juvenile development and egg hatch suppression.

The exceptional performance of *Trichoderma* enriched neem cake under pot and field circumstances is a glimmer in the nematology picture in the current study. The current findings were consistent with those of Affokpon^38^, who found that *T. asperellum* suppressed root-knot nematode and increased plant tolerance. *Trichoderma asperellum* biocontrol activity is attributed to the buildup of phytoalexins^39^. Increased plant growth of okra with *Trichoderma* enriched neem cake treated plants due to the reduction of root-knot nematode population. In comparison to untreated plants, treated plant roots absorb more nutrients from the soil. Several studies have shown that combining biocontrol agents with organic amendments increases plant growth and yield while also suppressing nematode populations^40–42^. However, there is a scarcity of information on the utilization of *T. asperellum* as a biocontrol agent against *M. incognita*. Present study showed that *T. asperellum* enriched neem cake can be used to control the root-knot nematode in vegetable crops. Nematode populations were found to be reduced in *Trichoderma* enriched neem cake compared to control. Plant parasitic nematodes are considerably reduced by neem and its derivatives^43,44^. The presence of toxic chemicals such as azadirachtin and nimbin (secondary metabolites) present in all regions of neem, which have a protective role against nematode infection and inhibited nematode proliferation^45^.

*T. asperellum* FbMi6 enriched neem cake improves plant tolerance in nematode-infected plants by boosting biochemical and physiological attributes such as polyphenols, total chlorophyll, nitrogen balance index, anthocyanin, and flavonoids as compared to control. The formation of secondary metabolites such as phenolic compounds hindered nematode reproduction^46^. Flavonoid inhibits nematode movement and egg hatching^47^, and plays an important function in plant defense^48^. Phenolic chemicals are produced in response to nematode stress in plants^49,50^. The amount of phenolic content accumulated in a plant determines its level of stress tolerance^51^. In comparison to the untreated inoculated control, use of bio-agents (*Pseudomonas aeruginosa*) in combination with neem cake produces systemic resistance in cotton^52^. Ammonia and higher the C/N ratio of organic inputs showed nematicidal action against plant parasitic nematodes. These substances may also affect root-knot nematode egg viability and hatching^53,54^. Organic additions alter the physiochemical and physical properties of the soil, which has a deleterious effect on nematode motility and host finding^55^. The similar technique may have worked against the root-knot nematode in okra. *Trichoderma* is a well-known, worldwide recognized biocontrol agent. Nonetheless, studies on *Trichoderma* spp. have focused on one or more parameters in a single strain. *T. asperellum*, the identified strain, has a quicker growth rate and a substantial nematocidal effects on *M. incognita*. According to our findings, pot and field studies showed that *T. asperellum* FbMi6 enriched neem cake considerably reduced the population of *M. incognita*. The remarkable effectiveness of *T. asperellum* FbMi6 enriched neem cake against root-knot nematode in outdoor circumstances was proved.

## Materials and methods

### Isolation and identification of bioagent

All methods were carried out in accordance with relevant guidelines and regulations. Wet soil samples were obtained from various places in Haryana, India. The location does not require any special permission. *Trichoderma* isolates were isolated on potato dextrose agar (PDA) culture plates with 50 μg mL^−1^ streptomycin from collected samples using the serial dilution method^56^ and culture was multiplied on potato dextrose broth (PDB) at 25 ± 2 °C in a BOD incubator.

The *Trichoderma* isolate was identified at the generic level based on morphological parameters such as growth pattern and colony colour. Genomic DNA was isolated in pure form from the culture plates. The ITS-rDNA partial gene was effectively amplified using primers ITS4 and ITS5. The ABI-BigDye^®^ Terminatorv3.1 Cycle Sequencing Kit was used to set up the sequencing PCR. Sequence generated was manually modified for uniformity using the ABI 3100 automated DNA sequencers. Further To determine identification, the sequence data was aligned with publicly accessible sequences and compared with the GenBank database using BLSATN. The sequences were aligned in ClustalX. The partial ITS-rDNA sequence was submitted to GenBank (NCBI) to get the accession number. MegaX software was used to construct a phylogenetic tree of related *Trichoderma* species through the maximum likelihood method^57^. This culture was given the name FbMi6 and sent to the National Fungal Culture Collection (NFCCI) in Pune, Maharashtra, India for storage (http://nfcci.aripune.org/).

### Maintenance of bio-agents

*Trichoderma asperellum* FbMi6, an isolate from the Department of Nematology at Chaudhary Charan Singh Haryana Agricultural University, Hisar, Haryana, India was kept and used in further research.

### Source of seeds

The seeds of brinjal (*Solanum melongena* L.) cv. Hisar Shyamal okra (*Abelmoschus esculentus*) cv. Hisar Unnat were procured from Department of Vegetable Sciences, CCS HAU, Hisar.

### Obtaining *M. incognita* eggs and second-stage juveniles

*Meloidogyne incognita* eggs and J_2_s were collected from a pure population kept on brinjal (*Solanum melongena* L.) cv. Hisar Shyamal at CCSHAU, Hisar, Haryana, India. The eggs were collected from infested brinjal roots using the sodium hypochlorite method^58^. The juveniles were extracted using cobb’s method^59^ followed by Modified Baermann Funnel Technique (MBFT)^60^.

### In vitro assay

Culture broth 100 mL was centrifuged for 20 min at 1500 rpm containing 1 × 10^8^ spores (CFU mL^−1^). Cell-free culture filtrates were recovered through 0.45 μm filters (Whatman™). Culture filtrate was checked for the presence of any fungal spores using PDA plating procedures. For Egg hatching inhibition assay, five surface sterilized egg masses were kept into tissue culture plates with four different concentrations at 20, 40, 60 and 80% of *T. asperellum* and distilled water and PDB were kept as control. The hatched juveniles were counted under a stereoscopic binocular microscope for alternate day upto 10 days and the percent hatching inhibition computed.

Juveniles’ mortality assay was conducted in tissue culture plates with five mL culture filtrates at four different concentrations (20, 40, 60 and 80%) with water and PDB as control. Hundred newly hatched J_2_ were transferred in each tissue culture plate well. Death of juveniles was examined at 24 h intervals up to 72 h under a stereo zoom binocular microscope (Gippon). The death of juveniles was confirmed after recovering in fresh water. All hatching and mortality assay were repeated twice and assay were conducted at room temperature (25 ± 2 °C).

### Screen house assay

One ton of neem cake (organic carbon—10.45%, nitrogen—0.84%, phosphorous—0.69%, Potassium—0.59%) was enriched with three liters of *T. asperellum* FbMi6 aqueous solution. Mixed biomass (*T. asperellum* and neem cake) was kept under shade for 15 days with a moisture content of 25–30% and temperature of 25–28 °C for proper enrichment. *T. asperellum* CFU were measured in neem cake after enrichment using a serial dilution procedure (56). Before sowing, experimental pots were filled with *T. asperellum* FbMi6 enriched neem cake. The experiment was conducted in earthen pots (15 cm diameter) in a screen house at CCSHAU Hisar, India (29°10' N; 75°46' E).

Treatments as: *T. asperellum* FbMi6 enriched neem cake (5 g kg^−1^ soil), *T. asperellum* FbMi6 enriched neem cake (10 g kg^−1^ soil), *T. asperellum* FbMi6 enriched neem cake (15 g kg^−1^ soil), *T. asperellum* FbMi6 enriched neem cake (20 g kg^−1^ soil), Carbofuran 3G (16 mg kg^−1^ soil) were applied respectively. Five replications of each treatment were used in a completely randomized design (CRD). Okra cv. Hisar unnat seeds were sowed, and one plant per pot was maintained after thinning. Freshly hatched 2000 J_2_s were inoculated in steam sterilized soil at sowing time. Hoagland solution was applied to plants^61^.

Plant height, fresh shoot and root weight, dried shoot and root weight of okra, nematode galls per plant, egg masses per plant, eggs per egg mass, second stage juveniles per 200 cc soil were measured at harvesting (45 days after sowing). Galls and egg masses were counted with the help of a hand lens and soil was processed by Cobb sieving method^59^.

After drying in a hot air oven, physiological and biochemical characteristics of okra were recorded. Folin–Ciocalteau assay was used to determine total phenolic content^62^. Oven dried 0.5 g samples were extracted in 10 ml ethanol (80%) and centrifuged at 10,000 rpm for 20 min. The supernatant was collected and evaporated until completely dry. Reagent FC (1 N) 100 µl and 20 µl of each extract were placed in test tubes and left for 8 min before adding 300 µl of sodium carbonate. For 30 min at 40 °C, the contents were allowed to incubate in the dark. At 765 nm, the absorbance was measured. On a dry weight basis, the phenolic content of the sample was reported in mg GAE/100 g of sample. Dualex sensor (Dualex^®^ Scientific sensor) was used to calculate total chlorophyll, NBI, anthocyanin, and flavonoid.

### Evaluation under field trial

At 15 days before sowing, field research plots (5 × 3 m^2^) were mixed with *T. asperellum* FbMi6 enriched neem cake (CFU 8 × 10^8^ ml^−1^). The experiment was carried out on okra (cv. Hisar unnat) at the CCS HAU in Hisar, Haryana, India. The initial population 235 J_2_ 200^–1^ cc soil was assessed.

Neem cake 1 ton ha^−1^, *T. asperellum* FbMi 6 enriched neem cake 1 ton ha^−1^, chemical check (33 kg Furadan 3G ha^−1^), and inoculated control were used in a Randomized Block Design (RBD) with five replications. Observations on root gall index and final nematode population were recorded at 90 days after seeding. Cobb's methods were used to assess the soil population^59^. Okra yield and root gall index^63^ were also measured.

### Statistical analysis

SPSS version 10.0 was used to analyze the data statistically. The means were separated using DMRT. Differences in treatment means were considered significant at *P* ≤ 0.05. Values with the different letter in a column are significantly different at *P* ≤ 0.05. SD was calculated from excel spreadsheet Ver. 16.

### Ethics approval and consent

In conducting this study, experiments on live vertebrates and/or higher invertebrates were not required any special permission for approval and consent. The variety of okra Hisar Unnat was developed by CCSHAU, Hisar therefore, it not required any permission. All images used in the manuscript are original.

## Supplementary Information

Supplementary Information.

## Data Availability

All data generated or analyzed during this study are included in this published article (and its Supplementary Information files). The sequence data obtained in this study are openly available in GenBank of NCBI at https://www.ncbi.nlm.nih.gov/ under the Accession No. MT529837.1.
