# Supplementary material for: The nematicidal potential of novel fungus, Trichoderma asperellum FbMi6 against Meloidogyne incognita
Source: Sci Rep. 2023 Apr 23;13:6603. doi: 10.1038/s41598-023-33669-z (PMC10123068; doi:10.1038/s41598-023-33669-z)
Supplement: Supplementary file 1 — Supplementary Information. [file 41598_2023_33669_MOESM1_ESM.docx]

Molecular identification of the fungal strain **6**.

PROCEDURE**:**

1. Genomic DNA was isolated in pure form, from the culture provided by the sender.
2. The **ITS-rDNA** partial gene was successfully amplified using primers ITS4 & ITS5.
3. The sequencing PCR was set up with ABI-BigDye® Terminatorv3.1 Cycle Sequencing Kit.
4. The raw sequence obtained from ABI 3100 automated DNA sequencer was manually edited for inconsistency.
5. The sequence data was aligned with publicly available sequences & analyzed to reach identity.

**Results of Molecular Identification:**

1. The tested fungal strain 6 showed 100.00 % sequence similarity with ***Trichoderma asperellum***.
2. Sequence analyses with NCBI accession number MT529837.1, *Trichoderma asperellum* clone SF_561 resulted in following alignment statistics.
3. Alignment statistics: Query Length - 543, Score - 980 bits (1086), Expect - 0.0, Identities – 543/543 (100%), Gaps - 0/543 (0%), Strand - Plus/ Plus

Query 1 ACCCAATGTGAACGTTACCAAACTGTTGCCTCGGCGGGGTCACGCCCCGGGTGCGTCGCA 60

||||||||||||||||||||||||||||||||||||||||||||||||||||||||||||

Sbjct 71 ACCCAATGTGAACGTTACCAAACTGTTGCCTCGGCGGGGTCACGCCCCGGGTGCGTCGCA 130

Query 61 GCCCCGGAACCAGGCGCCCGCCGGAGGAACCAACCAAACTCTTTCTGTAGTCCCCTCGCG 120

||||||||||||||||||||||||||||||||||||||||||||||||||||||||||||

Sbjct 131 GCCCCGGAACCAGGCGCCCGCCGGAGGAACCAACCAAACTCTTTCTGTAGTCCCCTCGCG 190

Query 121 GACGTATTTCTTACAGCTCTGAGCAAAAATTCAAAATGAATCAAAACTTTCAACAACGGA 180

||||||||||||||||||||||||||||||||||||||||||||||||||||||||||||

Sbjct 191 GACGTATTTCTTACAGCTCTGAGCAAAAATTCAAAATGAATCAAAACTTTCAACAACGGA 250

Query 181 TCTCTTGGTTCTGGCATCGATGAAGAACGCAGCGAAATGCGATAAGTAATGTGAATTGCA 240

||||||||||||||||||||||||||||||||||||||||||||||||||||||||||||

Sbjct 251 TCTCTTGGTTCTGGCATCGATGAAGAACGCAGCGAAATGCGATAAGTAATGTGAATTGCA 310

Query 241 GAATTCAGTGAATCATCGAATCTTTGAACGCACATTGCGCCCGCCAGTATTCTGGCGGGC 300

||||||||||||||||||||||||||||||||||||||||||||||||||||||||||||

Sbjct 311 GAATTCAGTGAATCATCGAATCTTTGAACGCACATTGCGCCCGCCAGTATTCTGGCGGGC 370

Query 301 ATGCCTGTCCGAGCGTCATTTCAACCCTCGAACCCCTCCGGGGGATCGGCGTTGGGGATC 360

||||||||||||||||||||||||||||||||||||||||||||||||||||||||||||

Sbjct 371 ATGCCTGTCCGAGCGTCATTTCAACCCTCGAACCCCTCCGGGGGATCGGCGTTGGGGATC 430

Query 361 GGGACCCCTCACACGGGTGCCGGCCCCGAAATACAGTGGCGGTCTCGCCGCAGCCTCTCC 420

||||||||||||||||||||||||||||||||||||||||||||||||||||||||||||

Sbjct 431 GGGACCCCTCACACGGGTGCCGGCCCCGAAATACAGTGGCGGTCTCGCCGCAGCCTCTCC 490

Query 421 TGCGCAGTAGTTTGCACAACTCGCACCGGGAGCGCGGCGCGTCCACGTCCGTAAAACACC 480

||||||||||||||||||||||||||||||||||||||||||||||||||||||||||||

Sbjct 491 TGCGCAGTAGTTTGCACAACTCGCACCGGGAGCGCGGCGCGTCCACGTCCGTAAAACACC 550

Query 481 CAACTTTCTGAAATGTTGACCTCGGATCAGGTAGGAATACCCGCTGAACTTAAGCATATC 540

||||||||||||||||||||||||||||||||||||||||||||||||||||||||||||

Sbjct 551 CAACTTTCTGAAATGTTGACCTCGGATCAGGTAGGAATACCCGCTGAACTTAAGCATATC 610

Query 541 AAT 543

|||

Sbjct 611 AAT 613

**Top five hits upon BLASTn analysis**

| **Gene Bank Accession No.** | **Description** | **Max score** | **Query cover** | **Query coverage** | **E value** | **Identity (%)** |
| --- | --- | --- | --- | --- | --- | --- |
| MT529837.1 | *Trichoderma asperellum* clone SF_570 | 980 | 980 | 100% | 0.0 | 100.00% |
| MT529422.1 | *Trichoderma asperellum* clone SF_561 | 980 | 980 | 100% | 0.0 | 100.00% |
| MT529370.1 | *Trichoderma asperellum* clone SF_146 | 980 | 980 | 100% | 0.0 | 100.00% |
| MT367901.1 | *Trichoderma asperellum* clone SF_94 | 980 | 980 | 100% | 0.0 | 100.00% |
| MT150599.1 | *Trichoderma asperellum* isolate UGM-LHAF | 980 | 980 | 100% | 0.0 | 100.00% |

**Ack Code- 3265-6, ITS Sequence**

**TTTACAACTCCCAAACCCAATGTGAACGTTACCAAACTGTTGCCTCGGCGGGGTCACGCCCCGGGTGCGTCGCAGCCCCGGAACCAGGCGCCCGCCGGAGGAACCAACCAAACTCTTTCTGTAGTCCCCTCGCGGACGTATTTCTTACAGCTCTGAGCAAAAATTCAAAATGAATCAAAACTTTCAACAACGGATCTCTTGGTTCTGGCATCGATGAAGAACGCAGCGAAATGCGATAAGTAATGTGAATTGCAGAATTCAGTGAATCATCGAATCTTTGAACGCACATTGCGCCCGCCAGTATTCTGGCGGGCATGCCTGTCCGAGCGTCATTTCAACCCTCGAACCCCTCCGGGGGATCGGCGTTGGGGATCGGGACCCCTCACACGGGTGCCGGCCCCGAAATACAGTGGCGGTCTCGCCGCAGCCTCTCCTGCGCAGTAGTTTGCACAACTCGCACCGGGAGCGCGGCGCGTCCACGTCCGTAAAACACCCAACTTTCTGAAATGTTGACCTCGGATCAGGTAGGAATACCCGCTGAACTTAAGCATATCA**


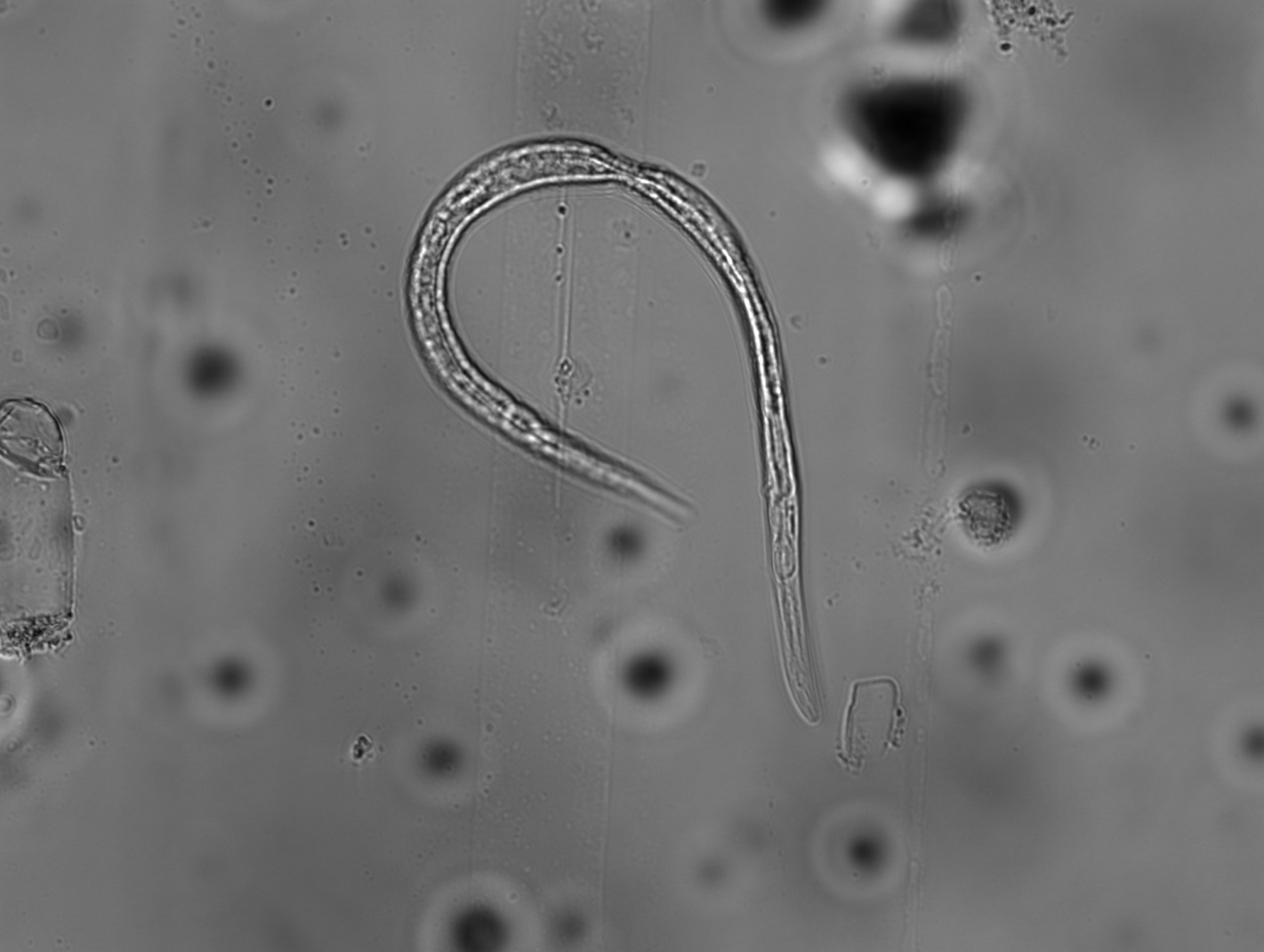


**Fig:** Mortality of root-knot nematode due to FbMi-4
